# Still in the shadows: a national study of acute mental health unit location across New Zealand hospitals

**DOI:** 10.1186/s12913-022-09004-z

**Published:** 2023-01-10

**Authors:** Anne Lian, Gawen Carr, Debbie Peterson, Gabrielle Jenkin, Helen Lockett, Susanna Every-Palmer, Ruth Cunningham

**Affiliations:** 1 Department of Public Health, University of Otago Wellington, Wellington, New Zealand; 2 Mental Health Addictions and Intellectual Disability Service, Capital and Coast DHB, Wellington, New Zealand; 3 Department of Psychological Medicine, University of Otago Wellington, Wellington, New Zealand; 4 WISE Group, Hamilton, New Zealand

**Keywords:** mental health services, accessibility, integrated care, severe mental illness, location, hospital services

## Abstract

**Background:**

This study aimed to explore the location of acute mental health inpatient units in general hospitals by mapping their location relative to hospital facilities and community facilities and to compare their proximity to hospital facilities with that of general medical acute units.

**Methods:**

We obtained Google maps and hospital site maps for all New Zealand public hospitals. Geographic data were analysed and mental health units’ locations in relation to hospital facilities and public amenities were mapped. Radar plots were constructed comparing acute medical and mental health units’ locations in relation to hospital facilities.

**Results:**

Twenty-two mental health units were identified. They were located predominantly at the periphery of hospital campuses, but also at a distance from community facilities. Compared to acute medical units, mental health units were almost universally located further from shared hospital facilities – with distances approximately three times further to reach the main hospital entrance (2.7 times distance), the nearest public café (3.4 times), the emergency department (2.4 times), and medical imaging (3.3 times).

**Conclusion:**

Despite the reforms of the 20^th^ Century, mental health units still appear to occupy a liminal space; neither fully integrated into the hospital, nor part of the community. The findings warrant further investigation to understand the impact of these structural factors on parity of health care provision between mental and physical health care and the ability of mental health care services to support recovery.

**Supplementary Information:**

The online version contains supplementary material available at 10.1186/s12913-022-09004-z.

## Background

Demand for mental health services has been rising around the world, with increased attention on the models of care required to meet this demand [1]. In New Zealand the population rate of contact with specialist services has risen by 10% between 2013 and 2021, while the proportion of adults who report unmet need for mental health care has nearly doubled between 2016/7 and 2021/22 [2, 3]. The 2018 Government Inquiry into Mental Health and Addictions found that services were not able to keep up with demand and made recommendations about expanding access and choice to services, particularly in the community, but was relatively silent on the continued role for hospital level specialist psychiatric care [4, 5]. However, the role of hospital care for acute mental health presentations has been an increasing focus of public and policy attention internationally [6]. In most Western countries, inpatient mental health units (MHUs) form a small but important part of the system for providing specialist acute mental health care. In New Zealand, publicly funded MHUs provide acute, short-term and sometimes involuntary care for approximately 14,000 people per year (approximately 8% of those cared for by specialist public mental health and addiction services, who are primarily cared for in the community) [3]. In order to design a fit for purpose mental health care system to meet increasing demand, consideration of roles of each part of the system is needed, including where each part of the system is best located in order to fulfil that role. This study focuses on acute mental health units as a small but critical part of the mental health care system.

Acute mental health units share a similar purpose to acute surgical or medical units in providing acute clinical care: an intense period of assessment, treatment and monitoring in a safe space, and continuous nursing [7]. In New Zealand, the care in acute inpatient units consists of psychiatric assessment and medication for symptoms of mental illness and distress, nursing, occupational therapy, social worker support and at times other forms therapy, with an increasing effort to provide access to cultural and peer support and other elements of holistic recovery focused care [8]. Once acute clinical needs have been met, care for both mental and physical health can then shift to a rehabilitation and community-based setting. While physical care has long been centralised to general or specialist hospitals to share common resources, mental health care has a much less integrated history. In New Zealand mental health care moved during the 1980s and 1990s from large standalone institutions to community-based care complimented by acute mental health care on hospital campuses but is commonly not well integrated into main hospital buildings [9].

Mental health and medical acute care require access to many of the same resources. Both need access to imaging and laboratory facilities, and easy access to consultation with other specialities along with rapid responses to medical emergencies [10]. Both also seek to avoid isolating patients, and therefore need to encourage family and friends to engage when clinically appropriate.. Acute mental health care also requires access to community resources such as cafés, libraries, green spaces and public transport as part of the acute assessment and treatment phase. For example, as stroke patients may need occupational therapy to assess a person’s home environment before discharge, acute mental health service users may need occupational therapy to assess and make plans for a person’s interaction with public transport and shops before discharge [11].

Access to clinical medical care is also critical in MHUs, with close integration with emergency departments and medical wards. This is due to the need to diagnose physical conditions presenting with psychiatric symptoms (such as hypothyroidism or encephalitidies), acute physical complications of mental illness (such as overdose, self-harm). and ongoing oft missed or under-treated chronic physical conditions which account for a large proportion of morbidity and mortality within this population [12]. Integrated mental health and physical health care, including colocation of services, is recognised as an important approach to address these unequal physical health outcomes experienced by people with severe mental health conditions [13].

As some of the resources needed for acute care are fixed geographically within hospitals, decisions as to where to locate MHUs will require a balancing between access to different types of resources in order to best meet the needs of the patient population. Some resources can be accessed remotely such as laboratories or specialist consultations, while some need physical proximity such as access to medical emergency response teams and community resources. After New Zealand decided to adopt the acute care model seen in physical health in the 1990s [14], colocation of MHUs with general hospitals became common place. This placement was limited by pre-existing hospital infrastructure and was sometimes isolated from community resources but had advantages in providing more integrated medical care [15] and potentially reducing stigma associated with “othering” of mental health care [16]. Having MHU located away from hospitals on their own site isolates units from general hospital medical facilities and their specialists, exacerbating existing physical health inequities, but can potentially increase access to important community resources [17, 18] and allow for location in more natural park-like therapeutic settings [8]. Building MHUs close to community resources can be difficult, with this often contested by locals and subject to NIMBYism [19].

Parity between acute physical and mental health admissions also needs to be considered in the location of acute mental health services and will be soon legally mandated by the Pae Ora Act [20]. While physical acute care in general hospitals often provides holistic care with hospital chaplains, hospital cafés, availability of allied health and onsite access to consult liaison psychiatric support, mental health acute care needs equal access to these resources. Visitors and family members also need equal access to their loved ones staying in mental health wards, with ease of access and reducing stigma being essential to recovery and social re-integration.

There has been limited investigation, and none to the authors’ knowledge in New Zealand, on the location of mental health units on hospital campuses. Comparing the location of mental health and general health units enables us to better understand how location impacts on access to resources needed for acute care and any differences which may impact on the ability of services to achieve parity and integration between mental and physical health care.

### Study aims:

- (1) To explore the siting of acute mental health inpatient units in New Zealand hospitals by mapping their location relative to hospital facilities and community facilities and amenities such as convenience stores and parks.
- (2) To compare proximity to hospital facilities between acute mental health and acute general medical units at the same hospitals.

## Methods

All adult acute inpatient mental health units (MHUs) across the country were identified from a list provided by the New Zealand Ombudsman. The entrance of the main adult acute inpatient MHU for each hospital was located using publicly available maps of hospitals and then this location was identified on Google Maps. Other locations of interest identified included: the main hospital entrance, acute general medical unit, emergency department entrance, medical imaging (radiology) department, spiritual centre, and public café. It was not possible to locate the exact entry point of medical units, radiology departments, public cafés or spiritual centres, and so the approximate middle point of these locations was used. Hospital maps with locations of interest were available online for all hospitals except Dunedin and Southland hospitals so maps were requested (and obtained) directly from hospital management.

Where there were several public cafés on one hospital campus, the one closest to the unit in question was chosen. For example, for the distance from the acute MHU to the café, the café closest to that unit was used, while for the distance from the medical unit to the café, the café closest to the medical unit was used. Where there were several acute medical units on a campus, the one closest to the main hospital entrance was selected. Where the acute MHU was on a different campus from the main hospital, the public cafés and spiritual centres on the MHU’s campus were identified if they existed. In these cases, the acute medical unit, emergency department, medical imaging department and main entrance of the corresponding main hospital campus were also identified.

### Measures

Two sets of measurements were taken. The first set were to measure the distance of the MHUs from both the community and the hospital facilities.

The second set of measures sought to compare the distances of the MHUs with the Medical units in terms of: distance from (A) medical imaging, (B) main entrance, (C) hospital public cafés, and (D) emergency department.

#### Community facilities

Public amenities (bus stop, dairy/convenience store, park and public café) close to each MHU were identified. Bus stops^1^ were identified as marked on Google Maps, supplemented by local council transport maps where needed. For the other amenities, searches were undertaken for "dairy"*^2^ or "convenience", “park” or “reserve”, and “café” and the nearest search result selected.

The straight line (“as the crow flies”) distance in metres from the MHU entrance and the medical unit to each of the locations of interest was estimated using Google Maps “measure distance” function. This involved right clicking on the location of interest as accurately as it could be identified on Google Maps, selecting the “measure distance” option and clicking on the location to be measured to, generating a distance between the two points which was then recorded. For measurements to parks, the distance to the part of the green space marked “reserve” or “park” on Google Maps that was closest to the MHU entrance was measured. The maximum campus distance was estimated by measuring the longest straight-line distance from one end of the hospital campus (as marked in pink on Google Maps) to the other.

#### Hospital facilities

For hospital locations of interest and public amenities which might be used by those being treated in acute MHUs or their families, the distance from the MHU entrance in metres is presented on radar plots, where the centre of the plot is the entrance to the acute MHU.

#### Comparisons

The distances from the acute MHU entrances to hospital locations were also compared with distances from the acute general medical units to these locations. These comparisons are presented on radar plots, where the centre of the plot represents the main entrance to the MHU and medical unit respectively. Where the distance between two locations of interest was 0 or unmeasurable because they were in the same building and their separate locations could not be accurately identified, a distance of 30 m was used for plots and ratio calculations. Distances for hospitals where the acute MHU is located on a separate campus were not included in comparisons as including them would have distorted the figures, given the much longer distances involved.

It was not possible to estimate the actual route/walking distance between locations because of the lack of sufficiently detailed maps. Thus the “as the crow flies” distance was used. Where the MHU was located on a separate campus from the main hospital, the “as the crow flies” distance was supplemented with the walking and driving times as calculated by Google Maps.

Qualitative data was also gathered from Google Maps and hospital maps, including whether it appears necessary from the map to go outside to access the MHU from the main hospital entrance, whether the MHU was a freestanding building separate from other buildings on the hospital campus and whether it was in the line of sight of the main hospital entrance. Whether the MHU was located at the far edge of the hospital campus, and other services that were frequently located near MHUs were also noted.

## Results

Twenty-two publicly funded hospitals with acute adult inpatient MHUs were identified across New Zealand. Of these, two were located at a site distant to the main hospital in their respective city (Wakari mental health facility is 2.8 km from the general Dunedin hospital and Hillmorton mental health facility is 3.3 km from the general Christchurch hospital). The other 20 MHUs were all located on general hospital campuses. Table 1 shows the hospitals and units identified and their location and managing District Health Board (DHB).

**Table 1:** Hospitals with acute adult inpatient mental health facilities in New Zealand

Hospital	DHB	City	MHU Name	More than 400 beds
Whangārei	Northland	Whangārei	Tumanako	N
North Shore	Waitematā	Auckland, North Shore	He Puna Waiora	Y
Waitakere	Waitematā	Auckland	Waiatarau	N
Auckland	Auckland	Auckland	Te Whetu Tawera	Y
Middlemore	Counties Manukau	Auckland	Tiaho Mai	Y
Waikato	Waikato	Hamilton	Henry Bennett	Y
Tauranga	Bay of Plenty	Tauranga	Te Whare Maiangiangi	N
Whakatāne	Bay of Plenty	Whakatāne	Te Toki Maurere	N
Rotorua	Lakes	Rotorua	Whare Whakaue	N
Gisborne	Tairawhiti	Gisborne	Te Whare Awhiora	N
Taranaki Base	Taranaki	New Plymouth	Te Puna Waiora	N
Hawke's Bay	Hawke's Bay	Hastings	Ngā Rau Rākau	N
Whanganui	Whanganui	Whanganui	Te Awhina	N
Palmerston North	MidCentral	Palmerston North	Ward 21	N
Hutt	3DHB (CCDHB, Hutt Valley, Wairarapa)	Lower Hutt	Te Whare Ahuru	N
Wellington	3DHB (CCDHB, Hutt Valley, Wairarapa)	Wellington	Te Whare o Matairangi	Y
Nelson	Nelson Marlborough	Nelson	Wahi Oranga	N
Te Nīkau, Grey Hospital	West Coast	Greymouth	Manaakitanga	N
Hillmorton/Christchurch	Canterbury	Christchurch	Te Awakura	Y
Timaru	South Canterbury	Timaru	Kensington Centre	N
Wakari/Dunedin	Southern	Dunedin	Ward 9B and 9C	N
Southland	Southern	Invercargill	Southland Inpatient Unit	N

Acute MHUs were located in freestanding buildings separate from other buildings on the hospital campus in all but three hospitals. Of the 85% (n = 17) located in separate buildings but on the main hospital campus, four had internal connections to other hospital buildings enabling access to the main hospital entrance. The remaining 13 were in separate buildings that could only be accessed from the hospital main entrance by going outside. In only five hospitals could the MHU entrance be seen (in line of sight) from the main hospital entrance.

**Fig. 1:**
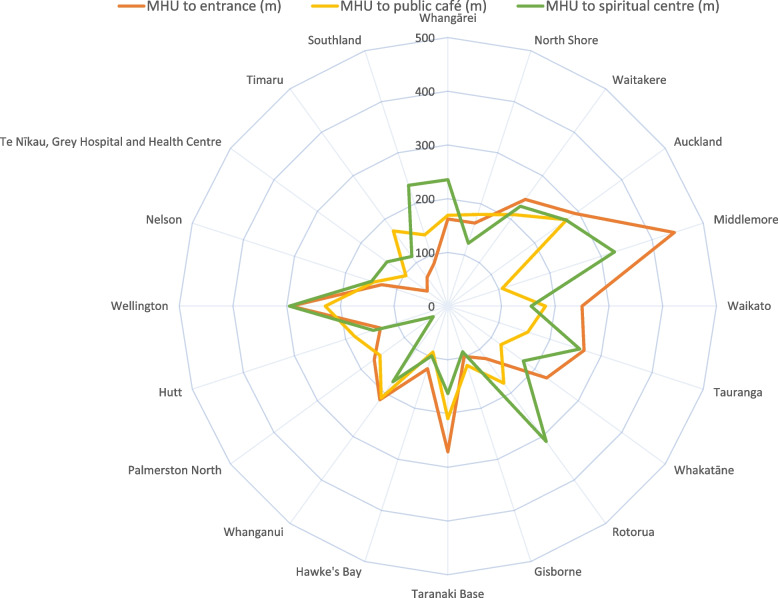
Distance from the Mental Health Unit (MHU) to patient and public facilities in the hospital (metres) in NZ hospitals

Figure 1 shows the location of acute MHUs in relation to patient and public facilities in the hospital (main entrance, public café and spiritual centre) among units located on main hospital campuses. The mean distance from the MHU to the main hospital entrance was 189 m, ranging from 48 m at Grey Hospital to 448 m at Middlemore hospital, New Zealand’s largest. The average distance from the MHU to the closest public café on the hospital campus was 166 m (ranging from 90 to 227 m) and from the MHU to the hospital spiritual centre the average distance was 186 m (ranging from 34 to 327 m). Seven of the mental health units (North Shore, Auckland, Middlemore, Whakatāne, Wellington, Waitakere and Whanganui) were located at the far edge of the hospital campus, often as far as possible from the main entrance. There was a notable difference between larger and smaller hospitals (as measured by bed capacity), with smaller hospitals tending to locate MHUs more centrally. Other services and locations noted to be frequently situated near acute MHUs included other mental health services (e.g. community, forensic, alcohol and drug and older adult services), dialysis units, rehabilitation units, administrative blocks and car parks.

**Fig. 2:**
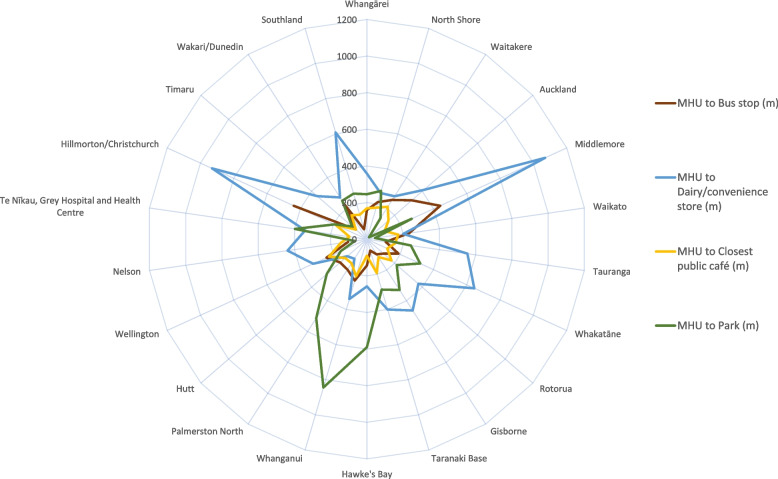
Distance from Mental Health Unit (MHU) to community facilities (in metres) in NZ hospitals

Figure 2 shows the location of acute MHUs in relation to community facilities. This figure includes the distances for the two MHUs located in separate mental health facilities away from the main hospital campus in Christchurch and Dunedin. Distances are also show in Supplementary Table 1. The distances to community facilities, particularly dairy or convenience stores and parks, is considerably further than the distances to hospital facilities. The average distance to a public bus stop was 196 m (ranging from 56 to 440 m). The average distance to a dairy or convenience store was 416 m (ranging from 127 to 1070 m). The average distance to a public café (which was most often the café located on the hospital campus) was 155 m (ranging from 81 to 228 m) and the average distance to a public park was 275 m (ranging from 15 to 844 m). One facility (Te Nikau) did not have any nearby public transport.

**Fig. 3:**
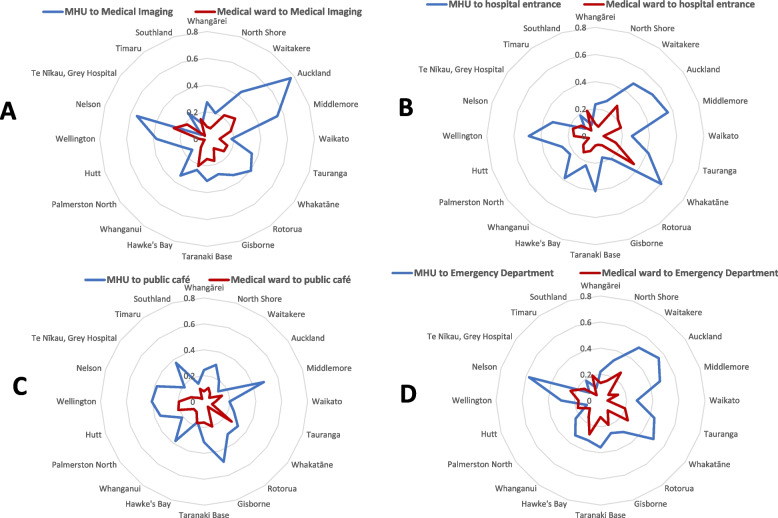
Distance to hospital facilities (**A**: Medical Imaging, **B**: hospital entrance, **C**: public café, **D**: Emergency Department) as a proportion of hospital campus size, comparing Mental Health Unit (MHU) and medical ward location

Figures 3 shows the comparison of the MHU location to that of an acute medical unit within the same hospital, as a proportion of the total hospital campus size, in relation to key hospital facilities (main entrance, medical imaging, emergency department and public café). For all the hospital facilities examined, the medical unit was closer than the MHU at almost all hospitals. Southland was the only hospital where the acute MHU was closer than the acute medical unit to all facilities. For the other 19 hospitals, the acute MHU was on average 2.7 times further from the main entrance, 3.4 times further from the nearest public café, 2.4 times further from the emergency department and 3.3 times further from the medical imaging department compared to the acute medical unit in the same hospital. Table 2 provides full details of distances from both MHU and acute medical units to hospital locations.

**Table 2:** Distances from mental health and medical units to hospital facilities

Hospital	MHU to entrance (m)	Med unit to entrance (m)	ratio	MHU to public café (m)	Med unit to public café (m)	ratio	MHU to spiritual centre (m)	Med unit to spiritual centre (m)	ratio
Whangārei	162.17	61.93	2.62	169.04	54.81	3.08	235.31	94.26	2.50
North Shore	162.45	43.4	3.74	179.25	66.15	2.71	123.2	30	4.11
Waitakere	245.4	140.67	1.74	210.48	113.72	1.85	229.65	103.37	2.22
Auckland	292.99	116.26	2.52	273.51	62.95	4.34	272.43	47.81	5.70
Middlemore	443.36	156	2.84	106.38	39.46	2.70	326.11	39.46	8.26
Waikato	249.74	56.8	4.40	181.83	71.61	2.54	154.97	97.2	1.59
Tauranga	266.53	102.3	2.61	156.44	41.68	3.75	258.01	110.88	2.33
Whakatāne	227.34	133.04	1.71	122.24	99.49	1.23	173.67	82.86	2.10
Rotorua	120.64	48.23	2.50	177.25	28.23	6.28	311.22	163.27	1.91
Gisborne	98.45	40.34	2.44	116.06	52.01	2.23	89.28	27.2	3.28
Taranaki Base	271.2	46.7	5.81	209.66	108.25	1.94	162.75	147.62	1.10
Hawke's Bay	122.64	62.58	1.96	90.11	94.81	0.95	97.24	92.61	1.05
Whanganui	215.32	82.62	2.61	211.08	55.24	3.82	173.95	24.7	7.04
Palmerston North	169.39	80.31	2.11	156.14	72.6	2.15	33.64	105.96	0.32
Hutt	131.79	25.51	5.17	182.31	107.46	1.70	145.76	53.9	2.70
Wellington	291.58	93.43	3.12	227.98	63.45	3.59	294.89	104.88	2.81
Nelson	129.36	67.85	1.91	144.89	30	4.83	149.26	30	4.98
Te Nīkau, Grey Hospital	47.56	66.24	0.72	96.59	24.81	3.89	139.89	30.8	4.54
Hillmorton/Christchurch*	3260	175.38	18.59	187.1	89.84	2.08	no spirit. centre	187.09	-
Timaru	65.75	17.99	3.65	172.77	71.61	2.41	114.25	39.83	2.87
Wakari/Dunedin*	2840	72.15	39.36	154.69	45.91	3.37	111.74	64.1	1.74
Southland	83.47	187.27	0.45	139.42	98.24	1.42	236.54	30	7.88

^*^Separate psychiatric and general hospital

The acute MHUs in Christchurch and Dunedin are located on separate campuses, approximately 19 and 39 times as far from the main hospital’s front entrance as the local acute medical units respectively. This represents a private vehicle driving time of 13 and 11 min respectively. The acute MHU in Christchurch was approximately 16 times as far from the emergency department and 38 times as far from the medical imaging department as the medical unit. For Dunedin, these values are 41 times and 36 times respectively.

## Discussion

In this study we aimed to explore the siting of acute mental health inpatient units relative to hospital facilities and community facilities and compared to acute general medical units, in order to understand the current state of service provision and its potential implications for care delivery. We found that acute MHUs were located at the periphery of general hospital campuses, far from most of the hospital facilities, but also far from community facilities such as shops, parks and public transport. Compared to acute medical units, acute MHUs were almost universally further from shared hospital facilities including the hospital entrance, the public café, the emergency department and radiology.

The location of MHUs at the periphery of hospitals may be understandable for hospitals built prior to 1990 which could not readily incorporate new MHUs within their current architecture and so were forced to choose between building on the hospital periphery or at an off-site location. However, there is no reason that this precedent should become the norm in hospital design, which appears to be what has happened, with recently builds either maintaining this peripheral position or not including mental health units at all (for example the new Christchurch and Dunedin hospital builds). Mental health units continue to occupy a liminal space; neither fully integrated into the hospital, nor part of the community.

There are potential advantages of either embedding acute MHU in a general hospital, or in a community outside of the hospital. The former may allow better medical care of complications and causes of mental disorder while reducing stigma within healthcare through normalising the inclusion of mental health alongside physical health care. The latter could potentially allow more space, better access to important community resources and the ability to increase visibility and potentially reduce stigma in communities. A community location may also enable closer connection to family/whanau and enable culturally embedded approaches for indigenous peoples such as Māori, the Indigenous people of New Zealand [21]. It is also possible that the location of units on the periphery of the general hospital campus could allow for the best of both worlds; there may be more space for bespoke architectural design catering to the model of care, with scope to relate buildings to the outdoor environment, and peripheral MHUs could be sited closer to community amenities like parks, shops and parking, while maintaining links to general hospital services.

However, our findings illustrate a system which may demonstrate the worst of both worlds—with potentially poor access to medical care through the barriers of distance, but also poor access to community facilities which support the first steps to community integration. This ongoing design choice in hospital architecture may entrench disparity in physical health outcomes over the life course by separating people needing acute mental health care from physical care. It may also result in increased shame and stigma experienced by patients when they are not given equal access to medical and community facilities. These finding are not in keeping with the current international and national guidelines and best practice recommendations and are contrary to the parity principles often enshrined in law [5, 10].

The location of MHUs away from other facilities may be said to shield occupants from the public gaze, reducing the ‘embarrassment’ of meeting acquaintances in the hospital. This idea reflects anachronistic attitudes toward mental illness – that it is shameful and should be kept out of sight. Many years of anti-discrimination campaigns around the globe are slowly changing perceptions of mental illness including the acceptability of receiving mental health treatment [22], but that is not the message given by mental health facilties that are kept out of sight. Moreover, the peripheral position can in fact place ward environments in public view: the stigma of acute mental health wards is not helped by features such as caged external fencing of courtyards in acute mental health facilities which being on the periphery of a hospital campus are often visible to passers-by. The peripheral placement of MHUs may also mean the maintenance and upgrade of these ‘out-of sight’ buildings are not equally prioritised as other hospital facilities. Certainly, in New Zealand, there has been considerable criticism of the physical state of many MHUs, with some described as not fit for purpose and in critical need of an upgrade [23].

This study provides a snapshot of metrics not often taken into consideration in health care facility design. It is not a marker of the overall quality of the MHUs. For example, the newest purpose-built MHU in NZ (Tiaho Mai at Middlemore Hospital) is sited on the periphery of the campus but has taken advantage of the available space to design a space that has been praised by staff and service users [24]. However, this study points to the trade-offs which may have been made in siting MHUs at the hospital periphery, particularly in light of the principle of parity between mental and physical health care included in the 2022 Pae Ora (Healthy Futures) Act [20].

Currently, the intention in New Zealand is to transform mental health and addiction services including overhauling many of the existing acute mental health inpatient facilities [25]. This includes exploring whether hospital sites are appropriate, or whether inpatient MHU could be replaced by smaller, community focused and embedded facilities. In order to make decisions about the appropriate location of care, a clear model of care is needed. This will involve co-design with service users and their family/whānau and thinking more holistically than just the bricks and mortar to consider how spaces and locations of care fit with the recovery model and connect people to their community. There is clear evidence in the current system of a lack of deliberate thought about how MHUs have historically been designed [8] – and, as shown in this study, where they are sited and how they relate to other health care facilities and the community.

This study used repeatable methods of examining publicly available Google Maps data combined with publicly available hospital map data. Google maps data are increasingly being used in research [26], including in mapping access to health care [27]. However, we are not aware of any other studies that consider within hospital location using public data in this way. Google maps data are based on satellite images and so have the advantage of direct correspondence to the real-world location of facilities. Every effort was made to identify each location of interest as accurately and consistently as possible on Google Maps, however the level of detail available on the hospital maps and sometimes their lack of correspondence to building footprints on Google Maps meant that individual measurements made will have some degree of inaccuracy. Moreover, the 2D nature of available maps and the ‘as the crow flies’ straight-line measurements taken do not capture the maze-like nature of hospital campuses with multiple storeys and complex sprawling lay outs. The actual navigated distances to facilities are therefore likely to be underestimated. Overall potential inaccuracies are unlikely to differ between mental health and medical hospital facilities and are likely to be small in magnitude relative to location differences uncovered in this paper. Therefore, the trends and patterns found are considered valid.

## Conclusions

Co-location of mental and physical health care in general hospitals occurred following the closure of separate psychiatric institutions and has the potential to both reduce stigma and improve integration of mental and physical health care. However, neither of these potential benefits is encouraged by the current location of mental health units in most of New Zealand’s hospitals.

Understanding where MHUs are currently located is the first step in considering the most appropriate location of these units. Consideration also needs to be given to the role inpatient services play in a predominantly community-based service and where they are best located to fulfil this role. As we attempt to achieve parity between physical and mental health care, and integration back into the community, we need to not only consider what services we need but where they are best located.

## Supplementary Information

**Additional file 1:** **Supplementary Table 1.** Distances from MHU main entrance to community facilities (m).

## Data Availability

The data used in this paper are publicly available via google maps and public hospital maps published online. The full derived dataset is presented in the paper.

## Footnotes

1 in all cases the closest public transport stops were bus stops.

2 “dairy” is the New Zealand word for a small owner-operated local grocery store.

## References

[1] WHO. Comprehensive mental health action plan 2013–2030. Geneva: World Health Organization; 2021. Available from: https://www.who.int/publications/i/item/9789240031029

[2] Ministry of Health. Mental Health and Addiction: Service Use 2019/20 tables 2021 [Available from: https://www.health.govt.nz/publication/mental-health-and-addiction-service-use-2019-20-tables.]

[3] Ministry of Health. Annual Update of Key Results 2021/22: New Zealand Health Survey. 2022 [Available at https://www.health.govt.nz/publication/annual-update-key-results-2021-22-new-zealand-health-survey]

[4] Government Inquiry into Mental Health and Addiction. He Ara Oranga: Report of the Government Inquiry into Mental Health and Addiction. Wellington; 2018. Available from: https://www.mentalhealth.inquiry.govt.nz/inquiry-report/. 10.1177/0004867419872810 31672046

[5] Allison S, Bastiampillai T, Castle D, Mulder R, Beaglehole B (2019). The He Ara Oranga Report: What’s wrong with ‘Big Psychiatry’ in New Zealand?. Aust N Z J Psychiatry.

[6] OECD. A new Benchmark for Mental Health Systems: Tackling the Social and Economic Costs of Mental Ill-Health. Paris: OECD Publishing; 2021.

[7] Bowers L, Chaplin R, Quirk A, Lelliott P (2009). A conceptual model of the aims and functions of acute inpatient psychiatry. J Ment Health.

[8] Jenkin GLS, McIntosh J, Every-Palmer S (2021). Fit for What Purpose? Exploring Bicultural Frameworks for the Architectural Design of Acute Mental Health Facilities. Int J Environ Res Public Health.

[9] 'Mental health services'. Te Ara. 2018. Available from: http://www.TeAra.govt.nz/en/mental-health-services. [Cited 3 Mar 2022].

[10] Australasian Health Infrastructure Alliance. Australasian Health Facility Guidelines Part B - Health Facility Briefing and Planning 134 - Adult Acute Mental Health Inpatient Unit. 2015. p. http://www.healthfacilityguidelines.com.au.

[11] Rugel E. Green Space and Mental Health: Pathways, Impacts, and Gaps. Vancouver: National Collaborating Centre for Environmental Health; 2015.

[12] Cunningham R, Peterson D, Sarfati D, Stanley J, Collings S (2014). Premature mortality in adults using New Zealand psychiatric services. NZMJ.

[13] Rodgers M, Dalton J, Harden M, Street A, Parker G, Eastwood A (2018). Integrated Care to Address the Physical Health Needs of People with Severe Mental Illness: A Mapping Review of the Recent Evidence on Barriers, Facilitators and Evaluations. Int J Integr Care.

[14] Thornicroft G, Tansella M (2004). Components of a modern mental health service: a pragmatic balance of community and hospital care: overview of systematic evidence. Br J Psychiatry.

[15] Naylor C, Das P, Ross S, Honeyman M, Thompson J, Gilburt H. Bringing together physical and mental health: A new frontier for integrated care. The King's Fund; 2016. 10.1177/0141076816665270 PMC5066536 27729592

[16] Verhaeghe M, Bracke P, Bruynooghe K (2007). Stigmatization in different mental health services: a comparison of psychiatric and general hospitals. J Behav Health Serv Res.

[17] Firth J, Siddiqi N, Koyanagi A, Siskind D, Rosenbaum S, Galletly C (2019). The Lancet Psychiatry Commission: a blueprint for protecting physical health in people with mental illness. The Lancet Psychiatry.

[18] Livingston JD (2020). Structural stigma in health-care contexts for people with mental health and substance use issues: A literature review.

[19] Cowan S (2003). NIMBY syndrome and public consultation policy: the implications of a discourse analysis of local responses to the establishment of a community mental health facility. Health Soc Care Community.

[20] Pae Ora (Healthy Futures) Act, 2022, sec. 7(1,e,iii) (New Zealand)

[21] Kopua MD, Kopua MA, Bracken PJ (2020). Mahi a Atua: A Māori approach to mental health. Transcult Psychiatry.

[22] Cunningham R, Peterson D, Collings S. Like Minds, Like Mine: Seventeen years of countering stigma and discrimination against people with experience of mental distress in New Zealand. In The Stigma of Mental Illness-End of the Story? Edited by W Gaebel, W Rössler & N Sartorius. Cham: Springer International Publishing; 2017. p.263–87.

[23] McNeilly H. 'That mixture should not happen': Ombudsman issues scathing report on psychiatric ward. https://www.stuff.co.nz/national/health/127665930/that-mixture-should-not-happen-ombudsman-issues-scathing-report-on-psychiatric-ward. 2022.

[24] Lewis O. New Zealand’s first ‘new wave’ mental health unit. https://www.newsroom.co.nz/new-zealands-first-new-wave-mental-health-unit. 2021.

[25] Ministry of Health. Mental Health and Addiction Specialist Services, Service Specifications (Tiers One, Two and Three). In: Ministry of Health, editor. Wellington, New Zealand2017.

[26] Rzotkiewicz A, Pearson AL, Dougherty BV, Shortridge A, Wilson N (2018). Systematic review of the use of Google Street View in health research: Major themes, strengths, weaknesses and possibilities for future research. Health Place.

[27] Weiss DJ, Nelson A, Vargas-Ruiz CA, Gligorić K, Bavadekar S, Gabrilovich E (2020). Global maps of travel time to healthcare facilities. Nat Med.

