# Supplementary material for: Still in the shadows: a national study of acute mental health unit location across New Zealand hospitals
Source: BMC Health Serv Res. 2023 Jan 10;23:21. doi: 10.1186/s12913-022-09004-z (PMC9830750; doi:10.1186/s12913-022-09004-z)
Supplement: Supplementary file 1 — Additional file 1: Supplementary Table 1. Distances from MHU main entrance to community facilities (m). [file 12913_2022_9004_MOESM1_ESM.docx]

Supplementary Table 1. Distances from MHU main entrance to community facilities (m)

| Hospital | Bus stop | Dairy/convenience store | Closest public café | Park |
| --- | --- | --- | --- | --- |
| Whangārei | 158.73 | 358.56 | 169.04 | 245.56 |
| North Shore | 211.39 | 262.9 | 179.25 | 275.16 |
| Waitakere | 255.12 | 278.71 | 210.48 | 136.63 |
| Auckland | 324.17 | 410.63 | 155.44 | 15.74 |
| Middlemore | 440 | 1070 | 106.38 | 268.71 |
| Waikato | 233.68 | 200.63 | 172.77 | 44.91 |
| Tauranga | 104.04 | 554.53 | 156.44 | 242.6 |
| Whakatāne | 189.91 | 644.86 | 122.24 | 320 |
| Rotorua | 120.06 | 371.67 | 177.25 | 215.47 |
| Gisborne | 98.45 | 462.7 | 116.06 | 329.63 |
| Taranaki Base | 64.67 | 399.98 | 192.33 | 287.21 |
| Hawke's Bay | 143.65 | 257.76 | 90.11 | 589.72 |
| Whanganui | 235.43 | 340.6 | 211.08 | 844.17 |
| Palmerston North | 197 | 127 | 156.14 | 512.03 |
| Hutt | 193.43 | 142.08 | 157.09 | 290.49 |
| Wellington | 242.76 | 322.69 | 227.98 | 157.09 |
| Nelson | 104 | 437.98 | 144.89 | 66.81 |
| Te Nīkau, Grey Hospital and Health Centre | No bus stop | 340.67 | 96.59 | 396.86 |
| Hillmorton/Christchurch | 439.62 | 930.85 | 187.1 | 194.55 |
| Timaru | 80.59 | 359.45 | 80.54 | 106.78 |
| Wakari/Dunedin | 223.9 | 270.85 | 154.69 | 249.47 |
| Southland | 56.46 | 607.86 | 139.42 | 259.62 |
| Mean distance | 197.9 | 418.8 | 154.0 | 276.4 |
